# Japan’s Nursing Workforce as Health System Security: From Silent Brain Drain to Brain Circulation

**DOI:** 10.31662/jmaj.2026-0053

**Published:** 2026-05-15

**Authors:** Kazumi Kubota, Kosuke Tsubota

**Affiliations:** 1 National Center for Child Health and Development, Tokyo, Japan; 2 Shimonoseki City University, Shimonoseki, Japan; 3 Department of Healthcare Information Management, The University of Tokyo Hospital, Tokyo, Japan; 4 The Graduate School of the International University of Health and Welfare, Tokyo, Japan

**Keywords:** nursing policy, brain drain, brain circulation, health workforce, reimbursement, Japan

## Abstract

Japan’s nursing policy has long been shaped by an implicit “closed-loop” assumption that language and licensure barriers will keep most nurses in a domestic labor market. Ten years after the Health Care 2035 vision was introduced, this assumption is weakening. Currency depreciation and widening international wage differentials, technology-enabled reductions in language-related preparation costs, and increasingly active global recruitment are together making outward mobility a realistic option for highly skilled nurses. Yet Japan lacks robust reintegration pathways for nurses who return with international education or experience, creating “brain waste” and accelerating loss of clinical leadership. This Opinion argues that nursing retention has become a national health system security issue directly affecting patient safety and system resilience rather than being a problem solvable within individual hospitals. Japan should pivot from ad hoc retention to an explicit “brain circulation” strategy, enabled by three policy levers: reimbursement mechanisms that make advanced nursing practice economically legible within the fee schedule, clinical-academic joint appointments that create landing zones for returnees, and scope-of-practice reform aligned with international standards.

## Introduction: Why Nursing Policy Is Health System Security

Japan’s Health Care 2035 vision invited Japan to pursue sustainability and global leadership ^(1)^. Yet the nursing workforce, one of the health system’s most important safety infrastructures, has often been treated as though it would remain structurally domestic. This assumption is becoming less reliable. This Opinion argues that Japan faces an emerging structural risk: not necessarily a dramatic, easily counted exodus, but a gradual thinning of high-skill nursing capacity and a persistent failure to reintegrate internationally trained nurses into roles that strengthen clinical care. The concern is precautionary rather than declarative: even before comprehensive emigration statistics are available, changes in mobility incentives and the underuse of returnees warrant policy attention because nursing capacity and work environments are closely linked to care quality and patient outcomes ^(2)^. When that happens, the consequences extend beyond staffing levels. A health system can replace headcount more readily than it can replace the people who train novices, stabilize high-acuity units, and lead day-to-day quality and safety work. In practical terms, if preceptorship and clinical leadership thin out in emergency care, intensive care, perioperative services, and complex chronic care, the system’s safety margin narrows even when staffing numbers appear stable.

**Figure 1.**
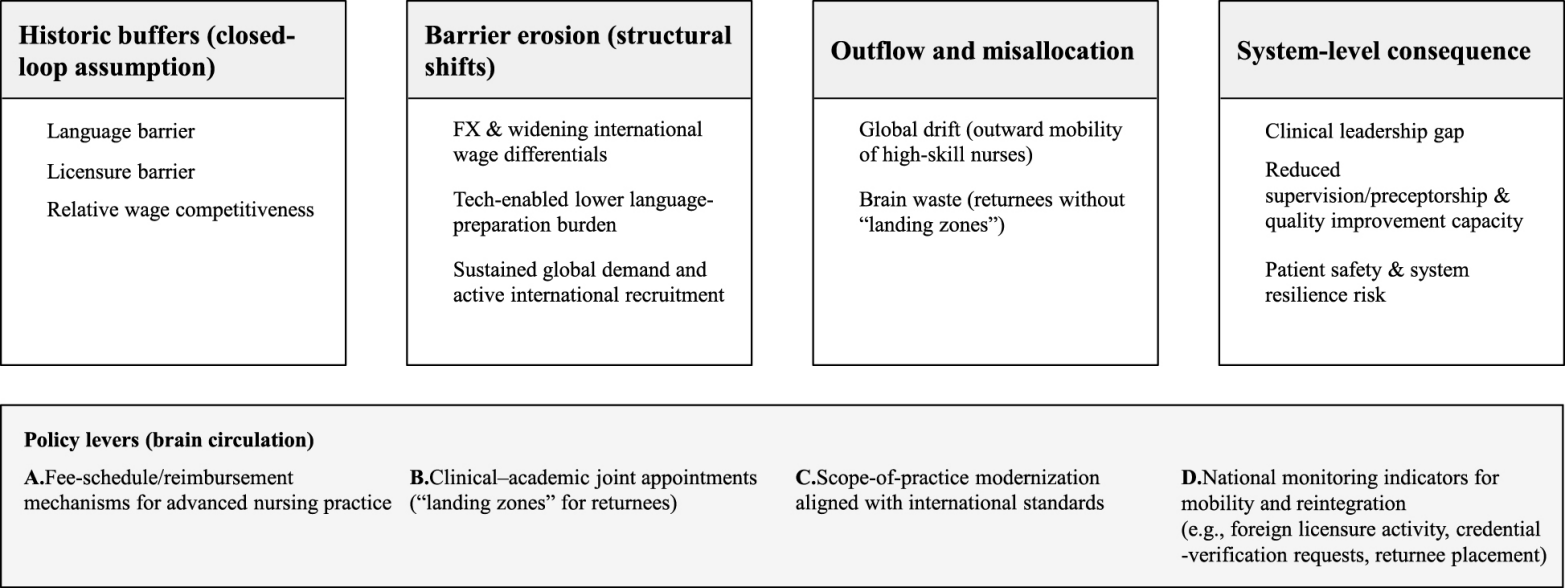
From “silent brain drain” to health system risk in Japan: where policy can intervene. A conceptual pathway showing how erosion of historical mobility buffers can lead to outflow and “brain waste,” creating a clinical leadership gap with implications for patient safety and system resilience. The figure highlights policy intervention points to shift from a retention-by-management to a brain-circulation strategy. Abbreviation: FX, foreign exchange.

The phrase “silent brain drain” is used here deliberately and cautiously. Japan lacks routine, transparent national statistics on nurse emigration, so the goal is not to claim precise volumes. Rather, the concern is that the decision environment for internationally mobile, high-aspiration nurses is changing in ways that are policy-relevant and, in principle, traceable through indirect indicators such as foreign licensure examination activity, credential-verification requests, destination-country registration data where identifiable, and bilateral visa or work-permit flows. Even modest outward movement from this segment may produce disproportionate system effects if it is concentrated in advanced clinical, supervisory, or precepting roles. Figure 1 summarizes the proposed mechanism and the policy intervention points.

## Eroding Mobility Buffers: Why the “Closed Loop” Is Less Reliable

Economic gradients are widening and becoming more salient. Organisation for Economic Co-operation and Development (OECD) indicators show substantial cross-national differences in remuneration and career pathways ^(3)^. Through currency depreciation, these differentials can feel larger and more immediate. Even if most nurses do not migrate, credible external options reshape expectations for a subset of the workforce, particularly for those who envision advanced practice, graduate education, or leadership roles. Japan’s challenge is not only wage level; it is wage responsiveness. The national fee schedule brings predictability and cost control, but it makes it difficult to render nursing expertise economically legible in ways that support durable clinical career ladders, protected time, or competitive compensation for advanced roles. Where advanced nursing practice can be linked to reimbursable services or recognized value streams, retention can be supported by structure, not only by local managerial effort.

Language-related frictions are also changing in practice. Technology does not remove the need for safe clinical communication or formal credentialing, and it should not be interpreted as a shortcut to competence. However, translation tools and artificial intelligence (AI)-assisted language support can reduce the preparatory burden for certain tasks and lower the cost of exploring overseas pathways ^(4)^. The policy implication is not that AI makes migration easy, but that it can make outward options more imaginable, and therefore more competitive, than they were under the closed-loop assumption.

Global demand is persistent and international recruitment is increasingly managed. World Health Organization (WHO) has emphasized that nurse mobility and migration must be monitored and responsibly managed because mobility patterns can reshape health systems ^(5)^. Japan is not operating in a passive environment. Even without making country-specific allegations about particular recruitment campaigns, the global context is enough: when high-income systems face sustained shortages, international recruitment pathways expand, and internationally trained nurses, especially those with transferable skills, become more mobile.

## The Preventable Failure Mode: “Brain Waste” on Return

Against this backdrop, Japan’s most preventable loss is not mobility itself, but “brain waste.” Nurses who obtain graduate education or specialized experience abroad often return to a domestic system that offers few structured landing zones that integrate advanced competencies into clinical practice. Some are treated as if they are starting over; others are forced into a binary choice between academia and bedside care with limited recognition of hybrid roles. The result is predictable: frustration, underutilization of skills, and eventual exit from clinical settings, sometimes followed by re-emigration. This is a system design failure. It converts potential brain circulation―mobility with return and knowledge transfer―into a one-way loss of clinical leadership.

From a system perspective, brain waste is costly because it disproportionately affects functions that support safe care under strain: mentoring and supervision, standardization of evidence-based practice, incident learning, and quality improvement. When these capabilities depend on a small number of individuals rather than institutional roles, they are brittle.

## Discussion: A Credible Brain-Circulation Strategy for Japan

Figure 1 functions as a policy map; here, “brain circulation” means accepting some outward mobility while designing incentives and reintegration pathways that return internationally acquired skills to patient care and protect core capacity. This should not be understood as a simple transfer of models from countries where diaspora networks, bilateral arrangements, or formal return-migration programs are more developed. For Japan, brain circulation is better framed as a domestically anchored reintegration strategy built around competency-matched return pathways.

A brain-circulation strategy must be credible within Japan’s governance and financing realities, and must be framed as a safety-oriented redesign of incentives, roles, and measurement rather than as a purely aspirational modernization agenda.

## Reimbursement As A Safety Instrument (Not Only A Payment Mechanism)

The first lever is to make advanced nursing practice economically legible within the fee schedule and related reimbursement mechanisms. This does not require importing another country’s model wholesale, nor does it require assuming that every advanced role must be independently billable.

In Japan, however, reimbursement change is not only a technical matter of payment design, but also a political and institutional one, requiring engagement among the Ministry of Health, Labour and Welfare, payers, hospital leadership, and professional bodies. A pragmatic starting point would be to recognize safety-sensitive functions, such as advanced consultation, complex care coordination, preceptorship, and quality-improvement responsibilities, within fee-schedule-constrained hospital financing, before pursuing broader reform. Without such mechanisms, hospitals remain structurally constrained: they are expected to retain advanced talent while lacking fiscal tools to support advanced roles and compensation.

Put simply, if policy expects hospitals to retain advanced nurses, it must provide at least one stable mechanism to plan and sustain advanced nursing roles within fee-schedule-constrained budgets.

## Reintegration Infrastructure: “Landing Zones” As National Capacity

The second lever is to institutionalize reintegration through clinical-academic joint appointments and other hybrid roles that function as landing zones for returnees. In Japan, these need not be assumed as a fully established national model; rather, they could be piloted within university hospitals and affiliated training networks as structured posts that combine clinical contribution with education, implementation, and research translation.

These positions allow internationally trained nurses to remain clinically grounded while contributing to education, research, implementation, and policy translation. Hybrid roles can strengthen preceptorship capacity, standardize evidence-based practice, and accelerate quality improvement in the places where care is delivered. Reintegration is consistent with WHO’s call to monitor and responsibly manage nurse mobility as a health-system governance issue ^(5)^.

Ethically, the question is not whether nurses move, but whether systems design pathways that protect patient welfare and professional dignity and avoid converting public investment in education into unreciprocated loss ^(6)^.

## Scope of Practice with Governance: Autonomy and Accountability Move Together

The third lever is modernization of scope of practice aligned with international standards, paired with explicit governance, training, and accountability. Regional experience in South Korea offers a nearby example that advanced practice legislation and role clarification can progress even under aging-related demand, financing constraints, and hierarchical professional cultures, although implementation remains politically contingent and cannot be transplanted wholesale to Japan ^(7)^. Not all outward mobility is motivated by the same factors. Some nurses may be drawn primarily by compensation differentials, while others may seek advanced specialty training, greater professional autonomy, or clearer clinical career ladders. This distinction matters because the policy response is unlikely to be uniform: pay and career sustainability may be central for one group, whereas recognized advanced roles and credible reintegration pathways may be more important for the other ^(8)^. Scope-of-practice reform is often framed as task shifting to reduce physician workload; this framing is incomplete. It is also a competitiveness strategy. High-aspiration clinicians do not leave only for salary; they leave to practice at the top of their competence and to see a future in which expertise is recognized. Any expansion of scope must therefore be designed to protect patients through clear role definitions, competency standards, and transparent responsibility for clinical decisions. Importantly, these changes should be introduced with nationally standardized competency benchmarks and evaluation, so that expanded roles strengthen, rather than dilute, clinical governance and patient safety.

## A Phased Approach to Improve Implementability

A simple way to avoid “grand reform” paralysis is to stage the strategy. Phase 1 should focus on interventions that do not require proving the magnitude of emigration to be justified: establishing national monitoring indicators, creating landing zones, and defining advanced clinical roles that directly support supervision, education, and safety. Phase 2 can then address more politically and administratively demanding reforms, including reimbursement refinement and broader scope-of-practice modernization.

## Limitations and Safeguards

This Opinion does not quantify outflow because Japan does not routinely publish comprehensive national statistics on nurse emigration. The aim of this opinion is to describe a policy-relevant mechanism and to identify low-regret monitoring and reintegration responses under uncertainty. WHO guidance supports treating mobility as a governance issue, while decisions remain multifactorial, and technology-enabled language support must not be overstated or used without safeguards ^(4), (5)^. Accordingly, the argument is framed as a precautionary policy case: even under uncertainty about magnitude, designing reintegration pathways and monitoring indicators is a low-regret strategy to protect patient safety and system resilience.

## Conclusion: Measure, Reintegrate, and Pay for Safety-Critical Expertise

A brain-circulation strategy requires measurement. Even if precise emigration counts are not routinely published, a minimum national dashboard is feasible and would improve accountability. At a minimum, it should include internationally comparable indicators of remuneration and career pathways; feasible proxies of outward mobility or outward intent, such as foreign licensure examination activity, credential-verification requests, and destination-country registration or visa data where traceable; reintegration measures capturing whether internationally trained nurses return to competency-matched clinical roles; and safety-sensitive indicators such as preceptor availability and turnover in high-acuity services. As a practical minimum, Japan can start by reporting a small set of indicators annually and publicly, then iteratively refine them as better data sources become available. The purpose is early detection and prevention.

Health Care 2035 challenged Japan to plan for the long term ^(1)^. The nursing workforce deserves the same structural foresight. Japan still has time to respond before a silent thinning of advanced capability becomes visible fragility. The choice is not between self-sufficiency and open borders. The choice is whether Japan designs policy that retains and recycles expertise through brain circulation, or continues to lose advanced capacity through avoidable brain waste, with predictable implications for patient safety and system resilience.

## Article Information

### Author Contributions

Kazumi Kubota and Kosuke Tsubota made substantial contributions to the conception and design of the work and to the interpretation of the evidence. Kazumi Kubota led the drafting of the manuscript. Kosuke Tsubota contributed to the policy framing and critically revised the manuscript for important intellectual content. Both authors approved the final version of the manuscript to be published, and agree to be accountable for all aspects of the work, ensuring that questions related to the accuracy or integrity of any part of the work are appropriately investigated and resolved. Kazumi Kubota and Kosuke Tsubota contributed equally to this work and share first authorship.

### Conflicts of Interest

None

### Ethics Approval and Consent to Participate

This article is an opinion paper and does not report any new studies with human participants or animals conducted by the authors. All ethical considerations discussed are based on previously published literature and established principles, including the Declaration of Helsinki.

### Statement on AI Use

In the preparation and revision of this manuscript, the authors used an AI-based language model (ChatGPT; OpenAI, San Francisco, CA, USA) to refine English phrasing and to improve structural coherence. The core concepts, policy arguments, revision decisions, and final review were performed by the authors, who take full responsibility for the integrity, accuracy, and interpretation of the work.
